# Supplementary material for: Girls’ Empowerment and Adolescent Pregnancy: A Systematic Review
Source: Int J Environ Res Public Health. 2020 Mar 4;17(5):1664. doi: 10.3390/ijerph17051664 (PMC7084341; doi:10.3390/ijerph17051664)
Supplement: Supplementary file 1 [file ijerph-17-01664-s001.zip › File S1.pdf]

Girl empowerment studies' critical appraisal

Cohort & Cross-sectional studies												
**National Institutes of Health (NIH) Quality Assessment tool for Observational Cohort and Cross-Sectional Studies**												
**STUDIES**	**Fagbamigbe 2012 [39]**			**Sarnquist 2014 [36]****			**Shrestha 2002 [40]**			**Vaz 2016 [37]**		
**Criteria**	Yes	No	Other (CD, NR, NA)*	Yes	No	Other (CD, NR, NA)*	Yes	No	Other (CD, NR, NA)*	Yes	No	Other (CD, NR, NA)*
1. Was the research question or objective in this paper clearly stated?	x				x		x			x		
2. Was the study population clearly specified and defined?	x			x			x			x		
3. Was the participation rate of eligible persons at least 50%?	x			x			x			x		
4. Were all the subjects selected or recruited from the same or similar populations (including the same time period)? Were inclusion and exclusion criteria for being in the study prespecified and applied uniformly to all participants?	x				x			x		x		
5. Was a sample size justification, power description, or variance and effect estimates provided?		x			x			x			x	
6. For the analyses in this paper, were the exposure(s) of interest measured prior to the outcome(s) being measured?	x			x			x				x	
7. Was the timeframe sufficient so that one could reasonably expect to see an association between exposure and outcome if it existed?	x				x		x					NA
8. For exposures that can vary in amount or level, did the study examine different levels of the exposure as related to the outcome (e.g., categories of exposure, or exposure measured as continuous variable)?	x			x			x			x		
9. Were the exposure measures (independent variables) clearly defined, valid, reliable, and implemented consistently across all study participants?			CD	x				x			x	
10. Was the exposure(s) assessed more than once over time?	x			x			x					NA
11. Were the outcome measures (dependent variables) clearly defined, valid, reliable, and implemented consistently across all study participants?	x			x			x			x		
12. Were the outcome assessors blinded to the exposure status of participants?			NA	x					NA			NA
13. Was loss to follow-up after baseline 20% or less?	x			x			x					NA
14. Were key potential confounding variables measured and adjusted statistically for their impact on the relationship between exposure(s) and outcome(s)?	x			x			x			x		
15. Overall judgement	HIGH			POOR			FAIR			FAIR		
**** CD: could not determine, NA: not applicable, NR: not reported***												

Qualitative Studies												
**Crtical Appraisal Skill Programme (CASP) tool for Qualitative Studies**												
**STUDIES**	**Lehman 2004 [33]**			**Lys 2012 [38]**			**Thorsen 2006 [41]**			**Juma 2013 [35]**		
**Guidelines**	yes	no	can't tell	yes	no	can't tell	yes	no	can't tell	yes	no	can't tell
Was there a clear statement of the aims of the research	x			x			x			x		
Is a qualitative methodology appropriate?			x	x			x			x		
Was the research design appropriate to address the aims of the research?	x			x			x			x		
Was the recruitment strategy appropriate to the aims of the research?			x	x				x		x		
Was the data collected in a way that addressed the research issue?		x		x			x				x	
Has the relationship between researcher and participants been adequately considered?			x	x					x	x		
Have ethical issues been taken into consideration?	x			x			x			x		
Was the data analysis sufficiently rigorous?	x			x			x			x		
Is there a clear statement of findings?	x			x			x			x		
How valuable is the research?	x			x			x			x		
Overall Judgement	LOW			HIGH			MODERATE			HIGH		
**Number in brackets next to study ID denotes the reference number in the main manucript's reference list												
