# Supplementary material for: Girls’ Empowerment and Adolescent Pregnancy: A Systematic Review
Source: Int J Environ Res Public Health. 2020 Mar 4;17(5):1664. doi: 10.3390/ijerph17051664 (PMC7084341; doi:10.3390/ijerph17051664)
Supplement: Supplementary file 1 [file ijerph-17-01664-s001.zip › File S2.pdf]

Systematic review

1. * Review title.

Give the working title of the review, for example the one used for obtaining funding. Ideally the title should state succinctly the interventions or exposures being reviewed and the associated health or social problems. Where appropriate, the title should use the PI(E)COS structure to contain information on the Participants, Intervention (or Exposure) and Comparison groups, the Outcomes to be measured and Study designs to be included.

Effect of girl's empowerment on adolescent pregnancy: a systematic review

2. Original language title.

For reviews in languages other than English, this field should be used to enter the title in the language of the review. This will be displayed together with the English language title.

3. * Anticipated or actual start date.

Give the date when the systematic review commenced, or is expected to commence.

01/12/2018

4. * Anticipated completion date.

Give the date by which the review is expected to be completed.

07/01/2019

5. * Stage of review at time of this submission.

Indicate the stage of progress of the review by ticking the relevant Started and Completed boxes. Additional information may be added in the free text box provided.
Please note: Reviews that have progressed beyond the point of completing data extraction at the time of initial registration are not eligible for inclusion in PROSPERO. Should evidence of incorrect status and/or completion date being supplied at the time of submission come to light, the content of the PROSPERO record will be removed leaving only the title and named contact details and a statement that inaccuracies in the stage of the review date had been identified.
This field should be updated when any amendments are made to a published record and on completion and publication of the review. If this field was pre-populated from the initial screening questions then you are not able to edit it until the record is published.

The review has not yet started: No

Review stage	Started	Completed
Preliminary searches	Yes	Yes
Piloting of the study selection process	Yes	No
Formal screening of search results against eligibility criteria	Yes	No
Data extraction	No	No
Risk of bias (quality) assessment	No	No
Data analysis	No	No

Provide any other relevant information about the stage of the review here (e.g. Funded proposal, protocol not yet finalised).

6. * Named contact.

The named contact acts as the guarantor for the accuracy of the information presented in the register record.

Chia-Ping Lin

Email salutation (e.g. "Dr Smith" or "Joanne") for correspondence:

Mr Lin

7. * Named contact email.

Give the electronic mail address of the named contact.

m536107001@tmu.edu.tw

8. Named contact address

Give the full postal address for the named contact.

250 Wuxing St. Taipei 11031, TW

9. Named contact phone number.

Give the telephone number for the named contact, including international dialling code.

+886 910594319

10. * Organisational affiliation of the review.

Full title of the organisational affiliations for this review and website address if available. This field may be completed as 'None' if the review is not affiliated to any organisation.

Taipei Medical University

Organisation web address:

http://www.tmu.edu.tw/main.php

11. * Review team members and their organisational affiliations.

Give the title, first name, last name and the organisational affiliations of each member of the review team. Affiliation refers to groups or organisations to which review team members belong.

Mr Chia-Ping LIN. Master program in Global Health and Development, Taipei Medical University
Mr Hexin, Latumer Katengeza. Master program in Global Health and Development, Taipei Medical University
Dr Dumisani Enricho Nkhoma. Master program in Global Health and Development, Taipei Medical University
Miss Estinfort Wanda. Master program in Global Health and Development, Taipei Medical University
Mr Charles Jenya Soko. Master program in Global Health and Development, Taipei Medical University

12. * Funding sources/sponsors.

Give details of the individuals, organizations, groups or other legal entities who take responsibility for initiating, managing, sponsoring and/or financing the review. Include any unique identification numbers assigned to the review by the individuals or bodies listed.

None

13. * Conflicts of interest.

List any conditions that could lead to actual or perceived undue influence on judgements concerning the main topic investigated in the review.

None

14. Collaborators.

Give the name and affiliation of any individuals or organisations who are working on the review but who are not listed as review team members.

Assistant/Associate Professor Usman Iqbal. Ph.D. & Master program in Global Health and Development, Taipei Medical University

15. * Review question.

State the question(s) to be addressed by the review, clearly and precisely. Review questions may be specific or broad. It may be appropriate to break very broad questions down into a series of related more specific questions. Questions may be framed or refined using PI(E)COS where relevant.

What is the effect of girl's empowerment (economic, education, policy, community, support) on adolescent pregnancy?

16. * Searches.

Give details of the sources to be searched, search dates (from and to), and any restrictions (e.g. language or publication period). The full search strategy is not required, but may be supplied as a link or attachment.

We will search the following electronic bibliographic databases: PubMed, Scopus, and Web of Science.

Using the following keywords: girl, women, female, empower, empowerment, economy, education, support, policy, community, and teenage, adolescent, pregnant, pregnancy. The search terms 'girl' or 'women' will be combined with 'empower' or 'empowerment'. 'Teenage' or 'adolescent' will be combined with 'pregnant' or 'pregnancy'. The keywords 'economic' or 'education' or 'support' or 'policy' or 'community' will be used to describe girl's empowerment and find the effect on adolescent pregnancy.

The search will be restricted for papers published in English only. This is the first review on this topic, therefore a wider search was warranted. Studies published between January 2000 and the date of searches were conducted will be included.

17. URL to search strategy.

Give a link to a published pdf/word document detailing either the search strategy or an example of a search strategy for a specific database if available (including the keywords that will be used in the search strategies), or upload your search strategy.Do NOT provide links to your search results.

https://www.crd.york.ac.uk/PROSPEROFILES/117414_STRATEGY_20181203.pdf

Alternatively, upload your search strategy to CRD in pdf format. Please note that by doing so you are consenting to the file being made publicly accessible.

Yes I give permission for this file to be made publicly available

18. * Condition or domain being studied.

Give a short description of the disease, condition or healthcare domain being studied. This could include health and wellbeing outcomes.

adolescent pregnancy(adolescent health), girl empowerment(global health)

19. * Participants/population.

Give summary criteria for the participants or populations being studied by the review. The preferred format includes details of both inclusion and exclusion criteria.

Inclusion: Definition of female adolescent according to WHO definition of adolescent 10 to 19 years

Exclusion: The studies that do not show clear association between girl empowerment (community, education, policy, support, economic) and adolescent pregnancy.

20. * Intervention(s), exposure(s).

Give full and clear descriptions or definitions of the nature of the interventions or the exposures to be reviewed.

In this review, the exposure will be empowerment in the form of education, economic, community and policy.

The outcome will be the effect of empowerment on adolescent pregnancy in our target population.

21. * Comparator(s)/control.

Where relevant, give details of the alternatives against which the main subject/topic of the review will be compared (e.g. another intervention or a non-exposed control group). The preferred format includes details of both inclusion and exclusion criteria.

Not applicable. The systematic review will include studies that clearly state the effect of girl empowerment on adolescent pregnancy.

22. * Types of study to be included.

Give details of the types of study (study designs) eligible for inclusion in the review. If there are no restrictions on the types of study design eligible for inclusion, or certain study types are excluded, this should be stated. The preferred format includes details of both inclusion and exclusion criteria.

All studies with the clear association between girl empowerment and adolescent pregnancy will be included regardless of the study design. These studies will also be peer-reviewed and full-text articles.

23. Context.

Give summary details of the setting and other relevant characteristics which help define the inclusion or

exclusion criteria.

There are no geographical restrictions in the systematic review.

24. * Main outcome(s).

Give the pre-specified main (most important) outcomes of the review, including details of how the outcome is defined and measured and when these measurement are made, if these are part of the review inclusion criteria.

The main outcome of the systematic review is the change (whether increase or decrease) in the number of adolescent pregnancy in the study population. These will be the study outcome and will be measured by the prevalence (baseline prevalence to prevalence after intervention) and incidence of adolescent pregnancy.

Timing and effect measures

Outcomes taken at any time following exposure to empowerment will be included.

25. * Additional outcome(s).

List the pre-specified additional outcomes of the review, with a similar level of detail to that required for main outcomes. Where there are no additional outcomes please state 'None' or 'Not applicable' as appropriate to the review

Effect on outcome by different types of empowerment or combination thereof.

Timing and effect measures

Outcomes taken at any time following exposure to empowerment will be included.

26. * Data extraction (selection and coding).

Give the procedure for selecting studies for the review and extracting data, including the number of researchers involved and how discrepancies will be resolved. List the data to be extracted.

All identified articles will be exported to EndNote citation manager (X9). Titles/abstracts of studies retrieved using the search strategy will be screened by five independent reviewers to identify studies that potentially meet the inclusion criteria outlined above. The full text of the eligible studies will be retrieved. Any disagreement among the reviewers regarding the eligibility of particular studies will be resolved through discussion with a sixth independent reviewer.

27. * Risk of bias (quality) assessment.

State whether and how risk of bias will be assessed (including the number of researchers involved and how discrepancies will be resolved), how the quality of individual studies will be assessed, and whether and how this will influence the planned synthesis.

The Cochrane risk of bias tool will be used to evaluate the risk of bias in the publications included

28. * Strategy for data synthesis.

Give the planned general approach to synthesis, e.g. whether aggregate or individual participant data will be used and whether a quantitative or narrative (descriptive) synthesis is planned. It is acceptable to state that a quantitative synthesis will be used if the included studies are sufficiently homogenous.

We will provide a narrative synthesis of the findings from the included studies structured around the type of

empowerment, population demographic and the effect of empowerment(s). We will provide summaries of effects on adolescent pregnancy from each studies.

29. * Analysis of subgroups or subsets.

Give details of any plans for the separate presentation, exploration or analysis of different types of participants (e.g. by age, disease status, ethnicity, socioeconomic status, presence or absence or co-morbidities); different types of intervention (e.g. drug dose, presence or absence of particular components of intervention); different settings (e.g. country, acute or primary care sector, professional or family care); or different types of study (e.g. randomised or non-randomised).

Provide details of plans to explore or analyze separately for different types of intervention (e.g.economic, education or the combination of both); different settings (e.g. high-middle-low income countries, geographic area); or different types of study (e.g. randomized or observational study).

30. * Type and method of review.

Select the type of review and the review method from the lists below. Select the health area(s) of interest for your review.

Type of review

Cost effectiveness
No

Diagnostic
No

Epidemiologic
No

Individual patient data (IPD) meta-analysis
No

Intervention
No

Meta-analysis
No

Methodology
No

Narrative synthesis
Yes

Network meta-analysis
No

Pre-clinical
No

Prevention
No

Prognostic
No

Prospective meta-analysis (PMA)
No

Review of reviews
No

Service delivery
No

Synthesis of qualitative studies

No

Systematic review
Yes

Other
No

Health area of the review

Alcohol/substance misuse/abuse
No

Blood and immune system
No

Cancer
No

Cardiovascular
No

Care of the elderly
No

Child health
No

Complementary therapies
No

Crime and justice
No

Dental
No

Digestive system
No

Ear, nose and throat
No

Education
No

Endocrine and metabolic disorders
No

Eye disorders
No

General interest
No

Genetics
No

Health inequalities/health equity
Yes

Infections and infestations
No

International development
Yes

Mental health and behavioural conditions
No

Musculoskeletal
No

Neurological

No

Nursing
No

Obstetrics and gynaecology
No

Oral health
No

Palliative care
No

Perioperative care
No

Physiotherapy
No

Pregnancy and childbirth
Yes

Public health (including social determinants of health)
Yes

Rehabilitation
No

Respiratory disorders
No

Service delivery
No

Skin disorders
No

Social care
No

Surgery
No

Tropical Medicine
No

Urological
No

Wounds, injuries and accidents
No

Violence and abuse
No

31. Language.

Select each language individually to add it to the list below, use the bin icon to remove any added in error.
English

There is an English language summary.

32. Country.

Select the country in which the review is being carried out from the drop down list. For multi-national collaborations select all the countries involved.

Taiwan

33. Other registration details.

Give the name of any organisation where the systematic review title or protocol is registered (such as with The Campbell Collaboration, or The Joanna Briggs Institute) together with any unique identification number assigned. (N.B. Registration details for Cochrane protocols will be automatically entered). If extracted data will be stored and made available through a repository such as the Systematic Review Data Repository (SRDR), details and a link should be included here. If none, leave blank.

34. Reference and/or URL for published protocol.

Give the citation and link for the published protocol, if there is one

Give the link to the published protocol.

Alternatively, upload your published protocol to CRD in pdf format. Please note that by doing so you are consenting to the file being made publicly accessible.

No I do not make this file publicly available until the review is complete

Please note that the information required in the PROSPERO registration form must be completed in full even if access to a protocol is given.

35. Dissemination plans.

Give brief details of plans for communicating essential messages from the review to the appropriate audiences.

Do you intend to publish the review on completion?

Yes

36. Keywords.

Give words or phrases that best describe the review. Separate keywords with a semicolon or new line. Keywords will help users find the review in the Register (the words do not appear in the public record but are included in searches). Be as specific and precise as possible. Avoid acronyms and abbreviations unless these are in wide use.

systematic review; female; girl empowerment; teenage; adolescent pregnancy; public health; International development

37. Details of any existing review of the same topic by the same authors.

Give details of earlier versions of the systematic review if an update of an existing review is being registered, including full bibliographic reference if possible.

38. * Current review status.

Review status should be updated when the review is completed and when it is published. For newregistrations the review must be Ongoing.
Please provide anticipated publication date

Review_Ongoing

39. Any additional information.

Provide any other information the review team feel is relevant to the registration of the review.

40. Details of final report/publication(s).

This field should be left empty until details of the completed review are available.

Give the link to the published review.
