# Supplementary material for: Girls’ Empowerment and Adolescent Pregnancy: A Systematic Review
Source: Int J Environ Res Public Health. 2020 Mar 4;17(5):1664. doi: 10.3390/ijerph17051664 (PMC7084341; doi:10.3390/ijerph17051664)
Supplement: Supplementary file 1 [file ijerph-17-01664-s001.zip › Figure S1.pdf]

Study ID	Experimental	Comparator	Outcome	Weight	Randomization process	Deviations from intended interventions	Missing outcome data	Measurement of the outcome	Selection of the reported result	Overall
Rodgers 2010	Preconception counseling	Usual care	Pyscho-social outcomes	1	?	?	–	?	–	–

+ Low risk
? Some concerns
– High risk

Figure S1: Risk of Bias assessment for randomized control trial done using Cochrane RoB tools ver.2.0 by Sterne, et al., [32].
